# Supplementary material for: A Cross-Sectional Study of Veterinarians in Germany on the Impact of the TÄHAV Amendment 2018 on Antimicrobial Use and Development of Antimicrobial Resistance in Dogs and Cats
Source: Antibiotics (Basel). 2022 Apr 5;11(4):484. doi: 10.3390/antibiotics11040484 (PMC9028039; doi:10.3390/antibiotics11040484)
Supplement: Supplementary file 1 [file antibiotics-11-00484-s001.zip › antibiotics-1653775-supplementary.pdf]

Freie Universität Berlin

Questionnaire

How does the TÄHAV amendment 2018 influence antimicrobial use and development of microbial resistance in dogs and cats?

Practice related questions

In what year did you start working as a practicing veterinarian? ___________

What is your status of employment?

☐ Practice owner
☐ Employee veterinarian

What size city do you work in?

☐ Small town	☐ Medium size city	☐ Large city
(< 20.000 residents)	(20.000 – 100.000 residents)	(> 100.000 residents)

What kind of facility do you work in?

☐ clinic ☐ practice

How many veterinarians are at your workplace?

☐ 1-2 ☐ 3-6 ☐ 7-10 ☐ more than 10

Approximately how many patients are treated per day?

☐ 10 – 20 ☐ 21 – 30 ☐ 31 – 50 ☐ more than 50

General antibiotic use

How many of your patients are treated with an antimicrobial daily?

☐ 0% - 10%
☐ 11% - 20%
☐ 21% - 30%
☐ 31% - 40%
☐ 41% - 50%
☐ 51% - 60%
☐ 61% - 70%
☐ 71% - 80%
☐ 81% - 90%
☐ 91% - 100%

Do you use topical or systemic antibiotics more frequently?

☐ Topical antibiotics
☐ Systemic antibiotics
☐ Both about equally often

Please answer the following questions using the matrix.

	Yes I fully agree	I rather agree	I partly agree	I rather dis-agree	No, I fully dis-agree
Penicillin´s are the antimicrobial group used most often.					
HPCIA (3rd/4th generation cephalosporins and fluoroquinolones) are rarely used antibiotics.					
HPCIA are used less frequent since the 2018 amendment to the TÄHAV.					
The TÄHAV amendment has led to a reduced use of antimicrobials in general.					
Since the introduction of obligatory antibiograms, more tests are being done.					
Due to the requirement of antimicrobial susceptibility testing, penicillins are generally used as the first line treatment.					
Penicillins must be changed particularly frequent after antimicrobial susceptibility testing.					

Do you request antimicrobial susceptibility tests more often right at the beginning of treatment or only when a change of antibiotic is necessary?

☐ Parallel to treatment

- ☐ Change of an antibiotic
- ☐ Both about equally often

For which diseases do you request antimicrobial susceptibility tests particularly often? (Multiple choice)

- ☐ Otitis
- ☐ Pyoderma
- ☐ Cystitis
- ☐ Wounds
- ☐ Diarrhea
- ☐ Respiratory infections
- ☐ Others _________________

For which animal species is antimicrobial susceptibility testing more often necessary?

- ☐ Dogs
- ☐ Cats
- ☐ Both about equally often

How do owners react to additional costs due to the antibiogram requirements?

- ☐ Understanding
- ☐ Rather understanding
- ☐ Partly understanding
- ☐ rather not understanding
- ☐ Not at all understanding

Specific diseases

<u>Otitis externa</u>

How often do you use antibiotic treatment with otitis externa?

- ☐ Always (80% - 100%)
- ☐ Frequently (60% - 79%)
- ☐ Partly (40% - 59%)
- ☐ Rarely (20% - 39%)
- ☐ Never (0% - 19%)

Do you use topical or systemic antibiotics more often to treat otitis externa?

- ☐ Topic antibiotics
- ☐ Systemic antibiotics
- ☐ Both about equally often

What active agents do you use most frequently for the treatment of otitis externa?

- ☐ Polymyxin B (Mitex/Surolan)
- ☐ Florfenicol (Osurnia/Neptra)
- ☐ Gentamicin (Easotic)
- ☐ Marbofloxacin (Aurizon)
- ☐ Others ________________

How often do you consult an antimicrobial susceptibility test for the treatment of otitis externa?

- ☐ Always (80% - 100%)
- ☐ Frequently (60% - 79%)
- ☐ Partly (40% - 59%)
- ☐ Rarely (20% - 39%)
- ☐ Never (0% - 19%)

Have you noticed an increased lack of efficiency of antibiotics during the treatment of otitis externa over the last 4 years?

- ☐ Yes
  - ☐ Polymyxin B (Surolan/Mitex)
  - ☐ Florfenicol (Osurnia/Neptra)
  - ☐ Gentamicin (Easotic)
  - ☐ Marbofloxacin (Aurizon)
  - ☐ Others _________________
- ☐ No

<u>Pyodermie</u>

How often do you use antibiotic treatment with pyoderma?

- ☐ Always (80% - 100%)
- ☐ Frequently (60% - 79%)
- ☐ Partly (40% - 59%)
- ☐ Rarely (20% - 39%)
- ☐ Never (0% - 19%)

Do you use topical or systemic antibiotics more often to treat superficial pyoderma?

- ☐ Topic antibiotics
- ☐ Systemic antibiotics
- ☐ Antiseptic treatment only

Do you use topical or systemic antibiotics more often to treat deep pyoderma?

- ☐ Topic antibiotics

- [ ] Systemic antibiotics
- [ ] Both parallel

What active agents do you use most frequently for the treatment of superficial pyoderma?

- [ ] Fusidic acid (Isaderm)
- [ ] Neomycin (Dermamycin Salbe)
- [ ] Polymyxin B (Surolan/Mitex)
- [ ] Others ______________

What active agents do you use most frequently for the treatment of deep pyoderma?

- [ ] Fluoroquinolones (Marbofloxacin, Enrofloxacin)
- [ ] Cephalosporins (Cefalexin)
- [ ] Penicillins (Amoxicillin, Amoxicillin - Clavulanic acid)

How often do you consult an antimicrobial susceptibility test for the treatment of pyoderma?

- [ ] Always (80% - 100%)
- [ ] Frequently (60% - 79%)
- [ ] Partly (40% - 59%)
- [ ] Rarely (20% - 39%)
- [ ] Never (0% - 19%)

Have you noticed an increased lack of efficiency of antibiotics during the treatment of pyoderma over the last 4 years?

- [ ] Ja
    - [ ] Fluoroquinolones (Marbofloxacin, Enrofloxacin)
    - [ ] Cephalosporins (Cefalexin)
    - [ ] Penicillin (Amoxicillin, Amoxicillin - Clavulanic acid)
    - [ ] Others ________________
- [ ] Nein

<u>Bissverletzungen</u>

How often do you use antibiotic treatment with bite wounds?

- [ ] Always (80% - 100%)
- [ ] Frequently (60% - 79%)
- [ ] Partly (40% - 59%)
- [ ] Rarely (20% - 39%)
- [ ] Never (0% - 19%)

Do you treat cat bites more often with a systemic antibiotic than dog bites?

- [ ] Yes

☐ No

What active agents do you use most frequently for the treatment of bite wounds?

☐ Penicillins (Amoxicillin, Amoxicillin-Clavulanic acid)
☐ Fluoroquinolones (Enrofloxacin, Marbofloxacin)
☐ Cefovecin (Convenia)
☐ Others ____________

How often do you consult an antimicrobial susceptibility test for the treatment of bite wounds?

☐ Always (80% - 100%)
☐ Frequently (60% - 79%)
☐ Partly (40% - 59%)
☐ Rarely (20% - 39%)
☐ Never (0% - 19%)

Have you noticed an increased lack of efficiency of antibiotics during the treatment of bite wounds over the last 4 years?

☐ Yes
  ☐ Penicillins (Amoxicillin, Amoxicillin-Clavulansäure)
  ☐ Fluoroquinolones (Enrofloxacin, Marbofloxacin)
  ☐ Cefovecin (Convenia)
  ☐ Others _______________
☐ No

<u>Cystitis</u>

How often do you use antibiotic treatment with cystitis?

☐ Always (80% - 100%)
☐ Frequently (60% - 79%)
☐ Partly (40% - 59%)
☐ Rarely (20% - 39%)
☐ Never (0% - 19%)

Do you treat cystitis in cats less often with an antibiotic than cystitis in dogs?

☐ Yes
☐ No

What active agents do you use most frequently for the treatment of cystitis?

☐ Penicillin (Amoxicillin, Amoxicillin – Clavulanic acid)
☐ Fluoroquinolones (Marbofloxacin, Enrofloxacin)

- [ ] Trimethoprim-Sulfonamid
- [ ] Others____________

How often do you consult an antimicrobial susceptibility test for the treatment of cystitis?

- [ ] Always (80% - 100%)
- [ ] Frequently (60% - 79%)
- [ ] Partly (40% - 59%)
- [ ] Rarely (20% - 39%)
- [ ] Never (0% - 19%)

What kind of sample do you use for antimicrobial susceptibility testing?

- [ ] Spontaneous urine
- [ ] Catheter urine
- [ ] Cystocentesis urine

Have you noticed an increased lack of efficiency of antibiotics during the treatment of cystitis over the last 4 years?

- [ ] Yes
    - [ ] Penicillin (Amoxicillin, Amoxicillin – Clavulanic acid)
    - [ ] Fluoroquinolones (Marbofloxacin, Enrofloxacin)
    - [ ] Trimethoprim-Sulfonamid
    - [ ] Others _______________
- [ ] No

Questions and comments on the questionnaire, antibiotic use, and the TÄHAV amendment.

____

____

____
